# Uni-portal non-coaxial spinal endoscopic surgery on percutaneous hollow screw fixation for atypical hangman's fracture: a novel minimally invasive technique case report

**DOI:** 10.3389/fsurg.2026.1871976

**Published:** 2026-07-28

**Authors:** Kai Luo, Fei Sun, Wenxian Huang, Haijun Tang, Mingxiu Yang, Wei Dai, Hongcai Teng, Shangyu Liu, Danting Xiao, Jianming Hu, Jingxin Deng, Haiyi Quan, Shengde Liang, En Song, Yun Liu

**Affiliations:** 1 Department of Orthopedics, Minzu Hospital of Guangxi Zhuang Autonomous Region, Nanning, China; 2 Department of Spine and Osteopathy, The First Affiliated Hospital of Guangxi Medical University, Nanning, China; 3 Orthopedics and Traumatology Department, Luliang County Traditional Chinese Medicine Hospital, Qujing, China; 4 Department of Sports Medicine, The First Affiliated Hospital of Kunming Medical University, Kunming, China; 5 Department of Plastic Surgery, The First Affiliated Hospital of Guangxi Medical University, Nanning, China

**Keywords:** atypical hangman's fracture, case report, minimally invasive, percutaneous hollow screw fixation, uni-portal non-coaxial spinal endoscopic surgery

## Abstract

**Purpose:**

This study reports a case of type B1 atypical Hangman's fracture and introduces a novel minimally invasive hollow pedicle screw insertion technique for the treatment of unstable Hangman's fracture.

**Methods:**

A 65-year-old male was admitted after an electric bicycle fall, presenting with occipital and cervical pain and numbness. Upon admission, his pain level was assessed as 7 on the Visual Analogue Scale (VAS), with a Neck Disability Index (NDI) score of 19. Cervical CT examination revealed multiple lucent lines in the axis, accompanied by diastasis, displacement, and fractures of the lateral transverse processes, leading to a diagnosis of type B1 atypical Hangman's fracture (AHF). The patient underwent percutaneous hollow pedicle screw fixation assisted by uni-portal non-coaxial spinal endoscopic surgery (UNSES).

**Results:**

Intraoperative blood loss was minimal, and postoperative CT confirmed satisfactory reduction of the axis fracture. At the 1-month follow-up, the patient's incision had healed well, symptoms had markedly improved (VAS score of 1, NDI score of 7). At the 10th-month follow-up, cervical CT showed significant fracture healing compared to previous examinations. By the 1-year follow-up, the patient's symptoms further improved (VAS score 0, NDI score 3).

**Conclusion:**

Percutaneous hollow pedicle screw fixation for AHF under the assistance of UNSES is feasible, offering advantages such as minimal invasiveness, a broad surgical field, and rapid postoperative recovery.

## Introduction

Hangman's fracture, also known as traumatic spondylolisthesis of the axis, is a common upper cervical spine injury. With the acceleration of modern life and changes in transportation methods, the incidence of hangman's fractures is on the rise. Hangman's fractures account for approximately 4%–7% of all spinal fractures and 20%–22% of axis fractures (1, 2). In 1985, Levine et al. (3) proposed a classification method that categorizes Hangman's fractures into four types—Type I, Type II, Type IIa, and Type III—based on the mechanism of injury, fracture morphology, and stability. Although the Levine-Edwards classification is widely applied in clinical practice and effectively guides treatment strategies, it does not include fracture types where the fracture line involves the posterior wall of the axis vertebral body. In 1993, Starr et al. (4) first reported a unique type of fracture involving a unilateral fracture line extending to the posterior wall of the axis vertebral body, which they termed atypical hangman's fracture (AHF). This discovery further deepened the understanding of hangman's fractures. Subsequent studies have further refined the classification of AHF. Scholars introduced additional subtypes, including A1 type, characterized by a fracture involving the posterior wall of the vertebral body on one side and the pars interarticularis (pedicle) on the opposite side; A2 type, involving the posterior wall of the vertebral body on one side and the articular process and/or lamina on the opposite side. For bilateral fractures, B1 type features fracture lines parallel to the posterior wall of the vertebral body, while B2 type involves one fracture line parallel and the other perpendicular to the posterior wall (1, 2, 5).

Stable Hangman's fractures are typically treated non-surgically, while unstable fractures, despite the absence of neurological symptoms in most patients, are generally managed with surgery to prevent secondary injury due to fracture instability. Compared to indirect anterior fixation, posterior pedicle fixation with hollow screws provides direct stabilization of the fracture, achieving immediate postoperative stability, and is widely used in clinical practice (6). Traditional open surgery for screw fixation, while providing a larger surgical field, involves extensive tissue dissection and carries a higher risk of nerve injury. With the advancement of minimally invasive techniques, some researchers have used robot-assisted or 3D fluoroscopy-guided methods to treat hangman's fractures, achieving precise screw fixation and significantly reducing tissue damage. However, these techniques also increase radiation exposure and operative time, and due to the high cost of robotic systems, they are not yet widely accessible (7–9). Therefore, finding a minimally invasive surgical technique with clear anatomical landmarks during the procedure and broader applicability for screw fixation remains an area of ongoing exploration for researchers.

Arthroscopic techniques have already been widely implemented in hospitals both domestically and internationally, and the necessary instruments are commonly available. We are the first to perform percutaneous hollow pedicle screw fixation for AHF under the assistance of uni-portal non-coaxial spinal endoscopic surgery (UNSES), achieving precise screw fixation and minimally invasive treatment. The effectiveness and safety of this technique have been confirmed in this case report. We hope that this approach provides spine surgeons with a new method for inserting hollow screws in the clinical treatment of AHF.

## Case presentation

**Figure 1:**
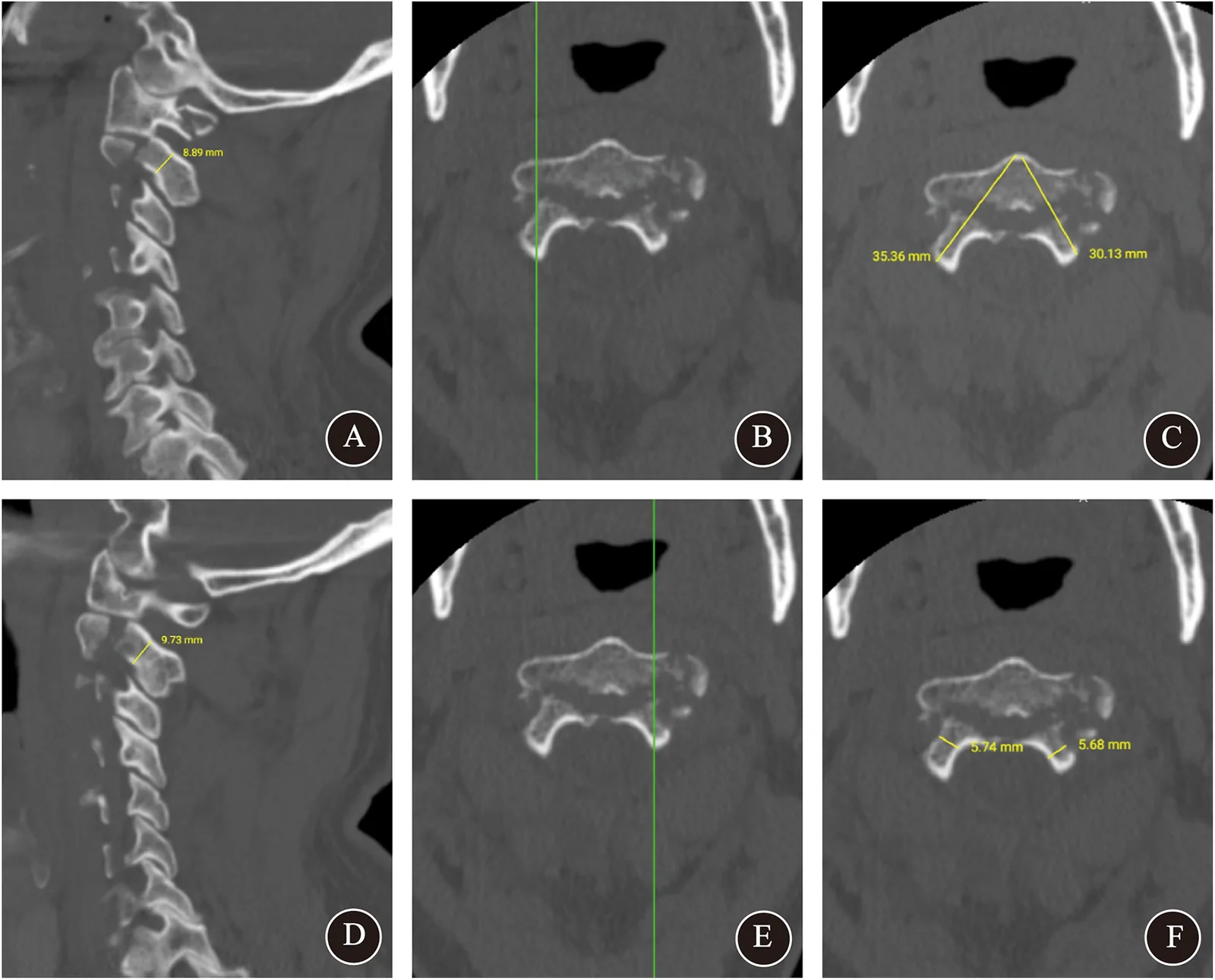
Preoperative cervical CT shows fractures of the right and left pedicles of the axis, with separation and displacement of the posterior wall of the axis. **(A,B)** The height of the right pedicle measures approximately 8.89 mm. **(C)** Preoperative planned measurement of pedicle screw length. **(D,E)** The height of the left pedicle measures approximately 9.73 mm. **(F)** The width measurements of the bilateral pedicles are 5.68 mm on the left and 5.74 mm on the right.

A 65-year-old male patient was admitted to the hospital after falling while riding an electric bicycle 1 day ago. The patient was described as suffering from numbness and pain in occipital and cervical regions [admission Visual Analogue Scale (VAS) score of 7, Neck Disability Index (NDI) score of 19] and grade Ⅴ muscle strength in both upper limbs. Cervical CT revealed multiple bright fracture lines in the axial vertebra and lateral transverse process, and the fractured end of the axial vertebra was separated and displaced, and the corresponding spinal canal was slightly narrowed (Figure 1). An atypical Hangman fracture (type B1) was diagnosed and the patient had no significant history of other infections, surgeries, or tumors. After completing preoperative preparations, we performed posterior percutaneous hollow pedicle screw fixation and reduction of the axis fracture under the assistance of UNSES.

### Surgical procedure and outcome

**Figure 2:**
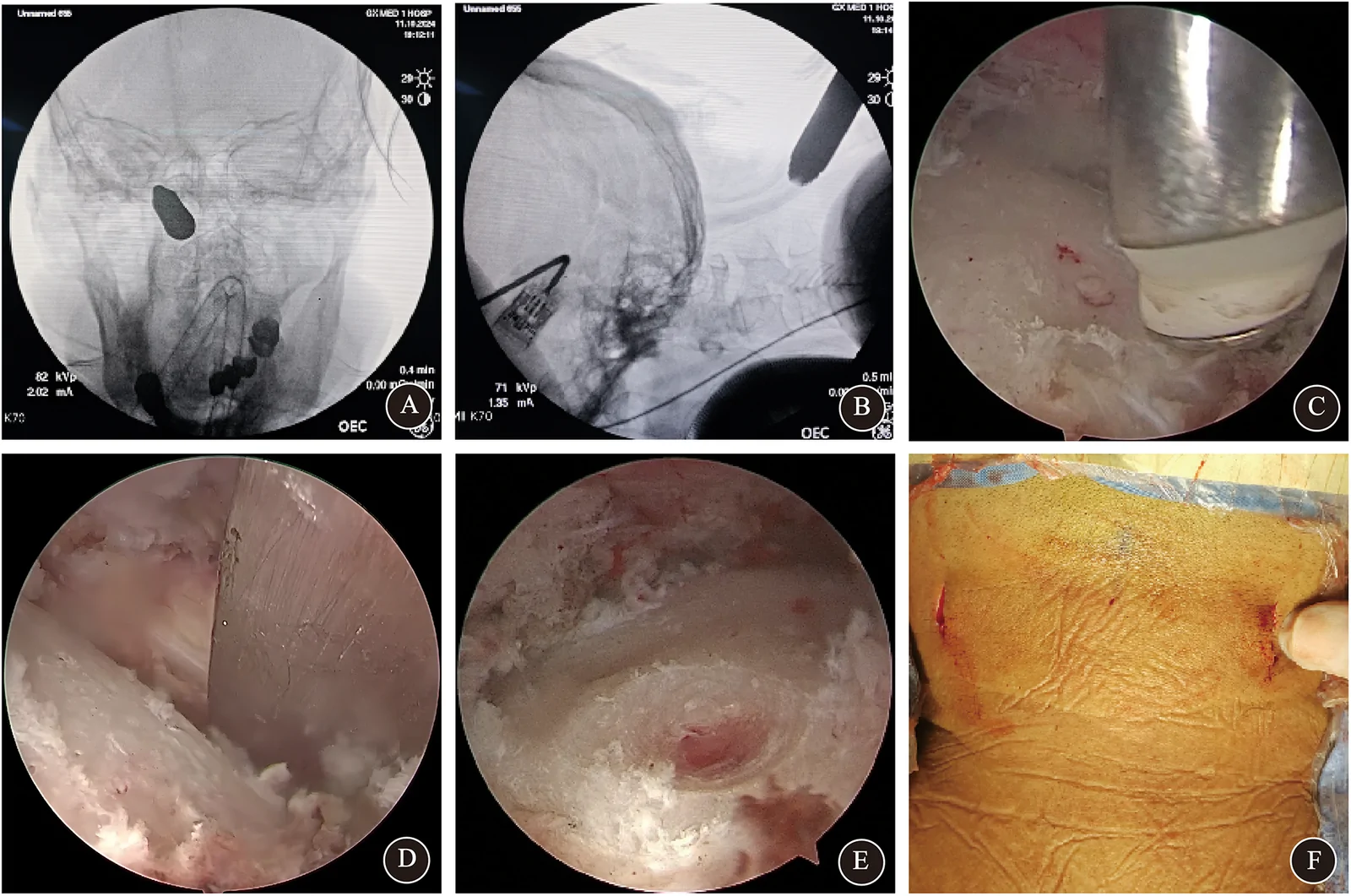
Intraoperative localization and endoscopic manipulation using the UNSES technique. **(A,B)** Intraoperative UNSES working channel positioning of the segment in the anteroposterior and lateral views under C-arm fluoroscopy. UNSES endoscopic intraoperative view. **(C)** Bonss high-frequency electrocautery being used to clear soft tissue around the C2 lamina. **(D)** Nerve dissector identifying the lateral margin of the C2 lateral mass. **(E)** Surgical burr marking the screw entry point. **(F)** Postoperative incisions measured approximately 1.8 cm on both sides.

**Figure 3:**
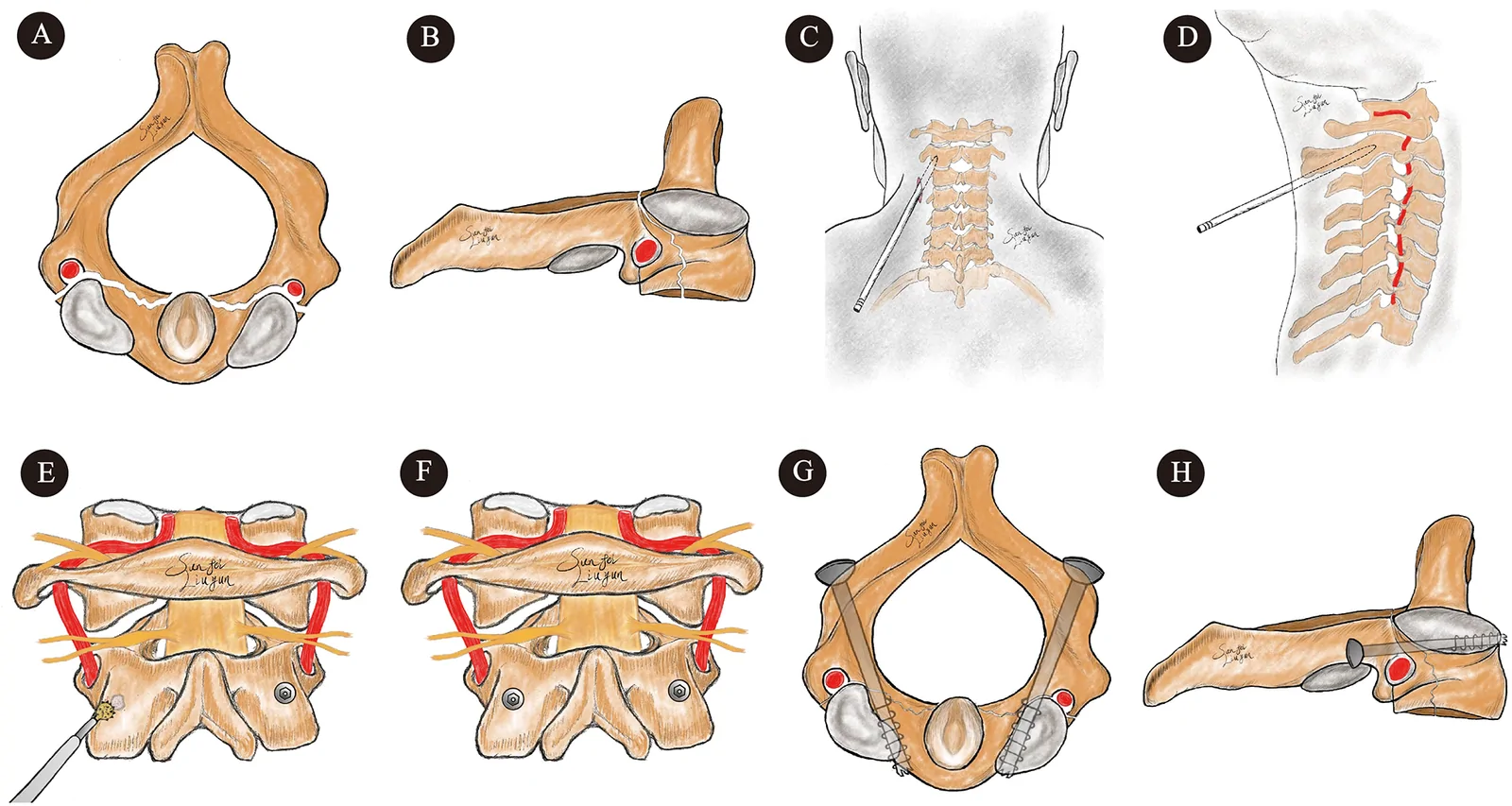
Illustration of UNSES-assisted percutaneous hollow screw fixation of AHF. **(A,B)** B1 type AHF diagram. **(C,D)** Use the smallest sequential dilators to locate the pivot. **(E)** The cortical bone at the screw insertion site was removed using the endoscopic burr, serving as a marker for screw placement. **(F,G,H)** The coronal, axial, and sagittal views after bilateral hollow screw insertion.

After general anesthesia, the patient was placed in the prone position, with Mayfield head fixation and continuous skull traction and continuous cranial traction applied. After routine disinfection and draping, a locating needle was first inserted approximately 3 cm lateral to the spinous process at a 25° downward and inward angle, with the tip directed toward the shoulder region of the atlantoaxial joint. After confirming satisfactory positioning under C-arm fluoroscopy, a 1.8 cm longitudinal incision was made along the locating needle. Sequential dilating cannulas were then inserted, and the surgical level was reconfirmed under C-arm guidance (Figures 2A,B). Subsequently, the endoscopic system was connected, and under UNSES guidance, the Bonss high-frequency electrocautery (Jiangsu Bonss Medical Technology Co., Ltd., Models 313 & 302) was used to dissect the paraspinal muscles, removing soft tissue from the lamina space to expose the atlanto-axial joint, the C2/3 facet joint, and the medial wall of the axis pedicle (Figures 2C,D). The midpoint of the upper and lower joint surfaces along the vertical line of the axis facet was chosen as the entry point for the hollow pedicle screw. The cortical bone at the screw fixation site was removed using an endoscopic burr (Figure 2E). A kirschner wire guide was then placed, followed by insertion of a kirschner wire. The endoscope was removed, and under C-arm fluoroscopic guidance, the kirschner wire was repositioned to confirm the angle and depth were correct. The screw length was measured, and a 4.0 mm × 30 mm hollow pedicle screw (Tianjin Jinxingda Industrial Co., Ltd. Partially threaded cannulated pedicle screw) was inserted on each side along the kirschner wire. Fluoroscopy confirmed satisfactory reduction of the fracture site, with no active bleeding observed. The incisions on both sides were approximately 1.8 cm each (Figure 2F). Finally, the skin was sutured and cranial traction was removed, and the technology is illustrated in Figure 3. The procedure lasted 150 min, involving only seven C-arm exposures and an estimated blood loss of 50 mL.

**Figure 4:**
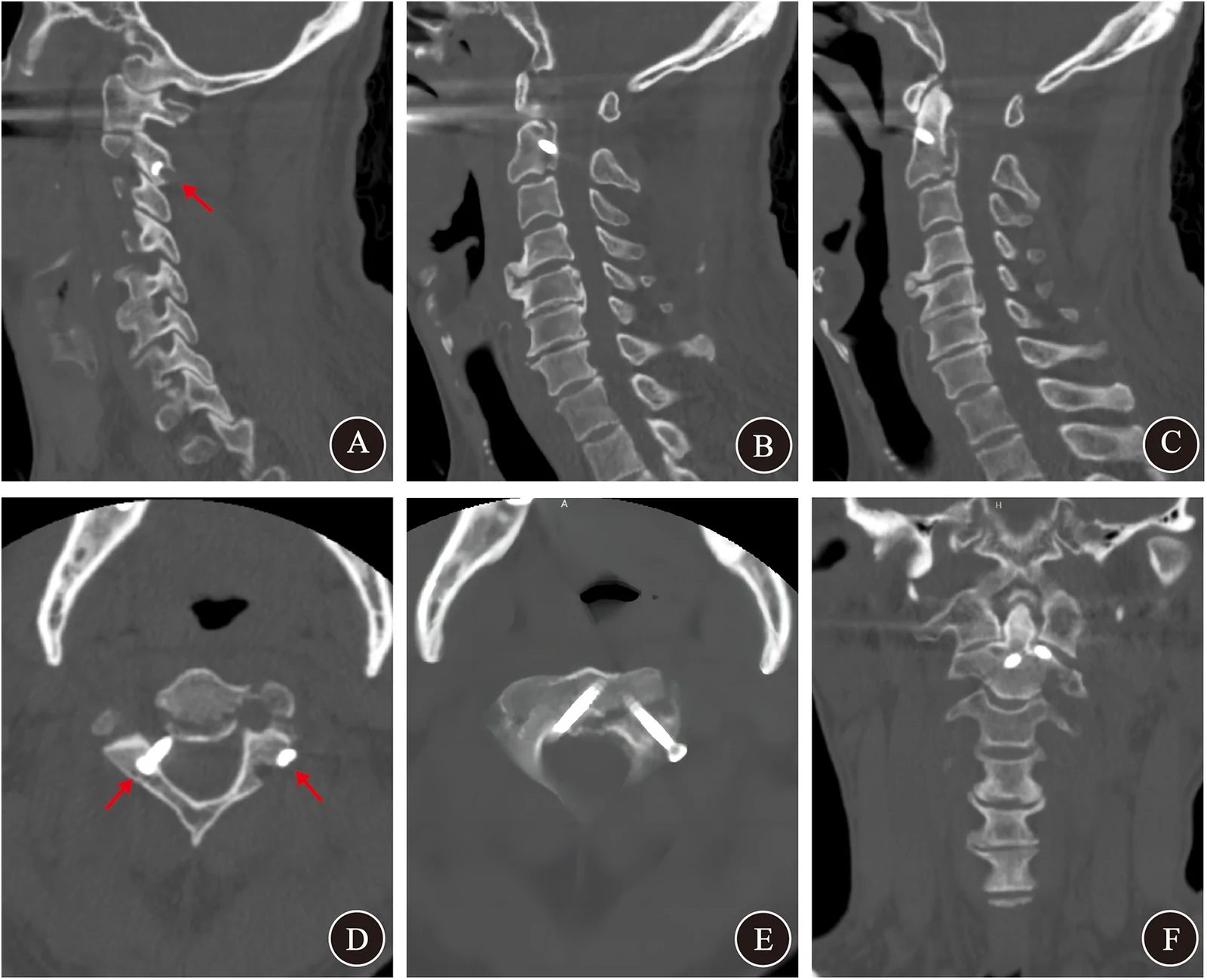
Postoperative cervical spine CT demonstrated accurate screw placement and satisfactory fracture reduction. **(A–C)** Sagittal views of the C2 cannulated screws, clearly showing the entry point (red arrow) and the trajectory of screw insertion. **(D,E)** Axial views of the C2 cannulated screws; panel D highlights the entry point (red arrow), while panel E demonstrates a significantly narrowed fracture gap compared to the preoperative status. **(F)** Coronal view of the C2 cannulated screws.

**Figure 5:**
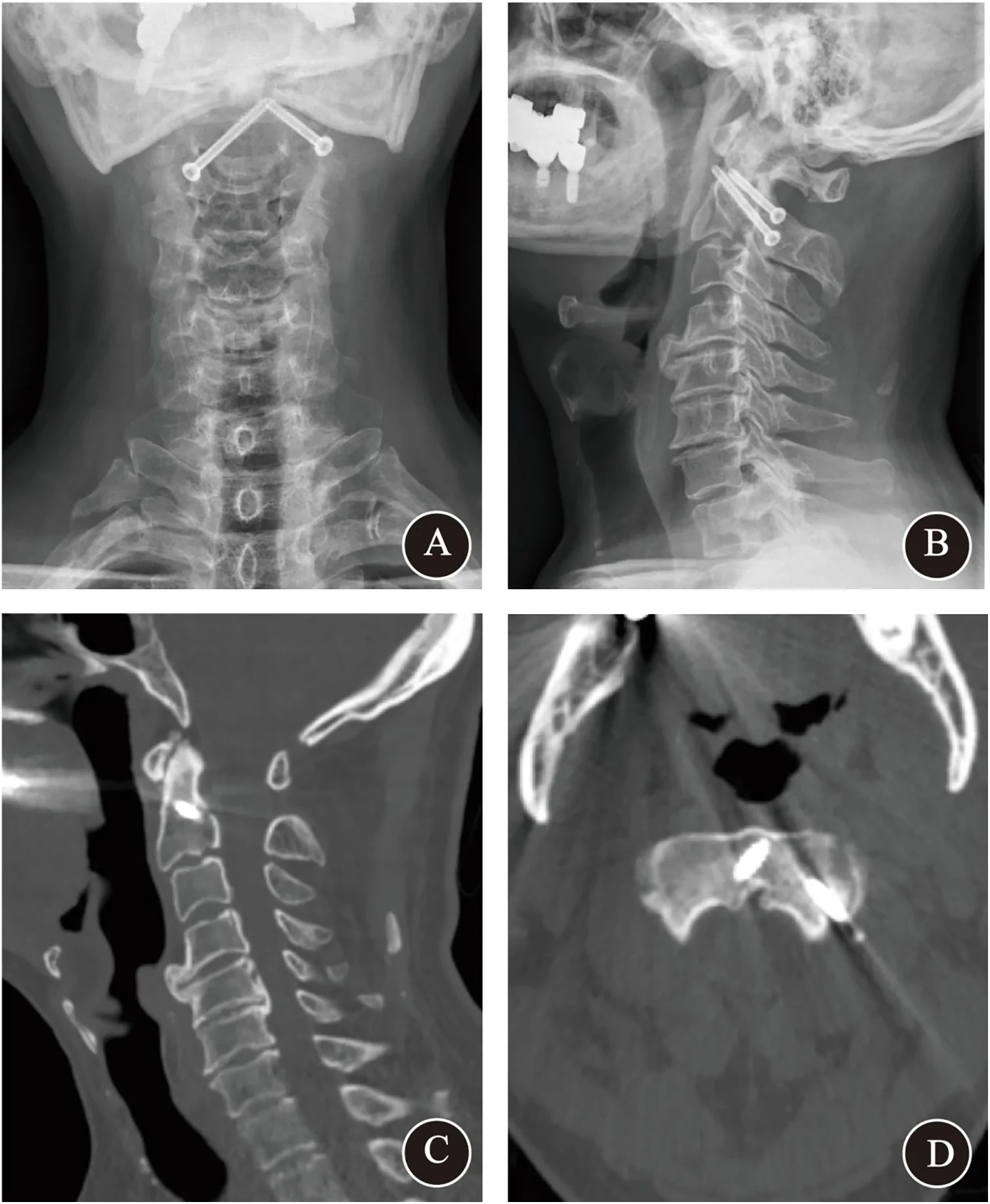
Follow-up cervical DR and CT images of the patient. **(A,B)** 1 month after surgery, cervical spine DR showing the proper positioning of the hollow pedicle screws. **(C,D)** 10 months postoperatively, cervical CT demonstrates significant fracture healing. Compared to previous examinations, the images reveal the presence of continuous bridging trabecular bone across the fracture site, accompanied by the disappearance of the fracture gap and restoration of cortical continuity.

Postoperative cervical spine CT showed that the screws were well positioned, with satisfactory fracture reduction. The screws engaged the anterior vertebral cortex without breaching it, thereby augmenting their pullout strength (Figure 4). According to the Gertzbein-Robbins scale, both pedicle screws were classified as Grade 2. On postoperative day 1, the patient was able to wear a neck brace and engage in limited activities. The patient reported a significant reduction in pain compared to preoperative levels (VAS score of 3, NDI score of 13). Intraoperative blood loss was minimal, and no drainage tubes were placed. The surgical wound dressings were dry. The patient was discharged on postoperative day 3. At the 1-month follow-up, the patient's incision had healed well, symptoms had markedly improved (VAS score of 1, NDI score of 7), and re-examination of the cervical vertebra by DR Showed that the screws were in good position (Figure 5A,B). At the 10 months postoperatively, cervical CT demonstrates significant fracture healing. Compared to previous examinations, the images reveal the presence of continuous bridging trabecular bone across the fracture site, accompanied by the disappearance of the fracture gap and restoration of cortical continuity (Figure 5C–D). By the 1-year follow-up, the patient's symptoms further improved (VAS score 0, NDI score 3).

## Discussion

AHF accounts for approximately 18%–74% of hangman's fractures (1, 10). Currently, surgical treatment is recommended for unstable fractures, with common surgical methods including anterior cervical discectomy and fusion, posterior pedicle screw fixation, posterior spinal fusion with pedicle screw fixation, and combined anterior-posterior fixation (11). The anterior approach involves critical structures such as the trachea, esophagus, carotid artery, and the superior laryngeal nerve, which increases the complexity and risk of the procedure. In this case, the fracture line of the axis vertebra is parallel to the posterior wall of the vertebral body, with bilateral pedicle fractures. The middle column structure is unstable and accompanied by neurological symptoms, classifying it as the B1 subtype of AHF. The anterior approach is difficult to maintain long-term stability, while the posterior approach is relatively simpler anatomically. The use of C2 hollow pedicle screws does not require intervertebral fusion and does not compromise cervical spine mobility, making it known as a “surgery that restores physiological function.” (12). Pedicle screws have an extremely low rate of loosening at the bone-screw interface and provide greater holding power, making them widely applicable in clinical practice. However, due to the complex anatomical structure of the cervical spine, which is closely adjacent to nerves and blood vessels, screw fixation still carries significant risks. Studies have pointed out that the narrowest point of the C2 pedicle is on average 5–7 mm. If a 3.5 mm diameter hollow screw is used to fix the fracture, the path within the pedicle becomes nearly the only viable route. If the initial screw fixation is not ideal, repositioning becomes very challenging. Furthermore, the medial side of the pedicle is adjacent to the cervical spinal cord, while the lateral and inferior aspects are near the vertebral artery foramen. Any injury to these structures could lead to severe complications (13–15). According to the literature, the risk of vascular and neurological injury during C2 pedicle screw fixation ranges from 11% to 66% (16). As a result, the precise placement of posterior pedicle screws has become a significant challenge for spine surgeons.

The choice of surgical technique is the key focus of this case report. Although traditional open posterior screw fixation provides clear anatomical landmarks and a larger surgical field, it involves significant tissue dissection and damage, resulting in considerable postoperative pain. This not only prolongs the recovery time but also lowers patient satisfaction. With the gradual application of minimally invasive techniques and new technologies in orthopedics, some researchers have proposed the use of computer-assisted methods for treating hangman's fractures. For instance, Li et al. (7) and Zhao et al. (8) used TINAVI robotic assistance to treat hangman's fractures. By performing intraoperative 3D scanning and planning the screw trajectory, this approach helps achieve more precise screw fixation, greatly reducing tissue damage, minimizing injury to adjacent small joints, lowering blood loss, and improving patient satisfaction postoperatively. Similarly, Zhao et al. (9) used computer-assisted navigation technology and found that both minimally invasive and open screw insertion could achieve precise screw fixation, with a first-grade screw placement rate as high as 87.5% (70/80). Barbagallo et al. (17) also reported a novel, minimally invasive technique using neuronavigation-assisted screw placement for atypical hangman's fractures. Although robotic-assisted and navigation techniques offer distinct advantages for C2 screw placement—utilizing relatively minimally invasive incisions to improve accuracy and safety—they necessitate additional incisions for the fixation of navigation reference frames, inevitably increasing iatrogenic trauma. Furthermore, these technologies rely heavily on intraoperative CT scans, which raises concerns regarding radiation exposure compared to our technique utilizing standard C-arm fluoroscopy. Additionally, the high cost of the robotic system, its maintenance costs, and longer scanning times are drawbacks that prevent many hospitals from adopting robotic surgery.

The UNSES technique, also known as arthroscopic-assisted uni-portal spinal surgery (AUSS), was first reported by Song et al. (18), which involves spinal surgery through a single, approximately 1.5–2.0 cm soft tissue incision with the assistance of an arthroscope. This approach offers advantages such as a wide field of vision, a large operative space, and strong compatibility with surgical instruments. It has been applied to various degenerative diseases, including cervical spondylosis, lumbar disc herniation, and spinal stenosis. In this case, the author first utilized the AUSS technique to assist in the screw fixation treatment of an atypical hangman's fracture. The use of AUSS to establish a minimally invasive screw insertion pathway, along with the application of high-frequency electrodes, not only reduces tissue damage and dissection but also fully exposes the fascial and muscular attachments to the deep lamina, achieving an “open surgical view.” The use of the grinding drill can remove the bone cortex of the screw insertion point, mark the screw insertion point and avoid the slide in the screw insertion process.

This technique enables minimally invasive screw insertion with clear visualization, achieving precise localization, significantly reducing the difficulty and risk of screw fixation, and minimizing radiation exposure to the patient. The instruments used in this technique are relatively standard, and no additional computer-assisted systems are required, making it a technique worth further promotion. Besides, the following points should be noted when using this technique: 1) The water pressure should be appropriately controlled to avoid any impact or damage to the spinal cord (below 40 mm Hg) (19); 2) The surgeon should be familiar with the cervical spine anatomy, ensuring that the arthroscopic view adequately exposes the upper edge of the axis lamina and the inner edge of the pedicle to better locate the screw fixation point; 3) The inserted hollow screws may reach the anterior cortical bone of the vertebrae, which can increase the screw's holding power and the stability of the vertebral body.

## Conclusion

We employed a novel technique to treat AHF—percutaneous hollow pedicle screw fixation with the assistance of UNSES. This report demonstrates the feasibility of this technique, which offers advantages such as minimal trauma, low blood loss, a wide field of vision, and rapid postoperative recovery. This technique provides spine surgeons with a new method for screw fixation in the treatment of AHF in the future.

## Data Availability

The datasets presented in this study can be found in online repositories. The names of the repository/repositories and accession number(s) can be found in the article/Supplementary Material.
