# Correction: Uni-portal non-coaxial spinal endoscopic surgery on percutaneous hollow screw fixation for atypical hangman's fracture: a novel minimally invasive technique case report

**DOI:** 10.3389/fsurg.2026.1973695

**Published:** 2026-09-08

**Authors:** Frontiers Production Office

**Affiliations:** Frontiers Media SA, Lausanne, Switzerland

**Keywords:** atypical hangman's fracture, case report, minimally invasive, percutaneous hollow screw fixation, uni-portal non-coaxial spinal endoscopic surgery

Affiliation Department of Orthopedics, Minzu Hospital of Guangxi Zhuang Autonomous Region, Nanning, China was omitted from the paper. This affiliation has now been added for author Kai Luo.

The corrected author list reads:

Kai Luo^1,2^, Fei Sun^3^, Wenxian Huang^2^, Haijun Tang^2^, Mingxiu Yang^2^, Wei Dai^2^, Hongcai Teng^2^, Shangyu Liu^2^, Danting Xiao^2^, Jianming Hu^2^, Jingxin Deng^2^, Haiyi Quan^2^, Shengde Liang^5^, En Song^4^*, Yun Liu^2^*

^1^Department of Orthopedics, Minzu Hospital of Guangxi Zhuang Autonomous Region, Nanning, China

^2^Department of Spine and Osteopathy, the First Affiliated Hospital of Guangxi Medical University, Nanning, China

^3^Orthopedics and Traumatology Department, Luliang County Traditional Chinese Medicine Hospital, Qujing, China

^4^Department of Sports Medicine, the First Affiliated Hospital of Kunming Medical University, Kunming, China

^5^Department of Plastic Surgery, the First Affiliated Hospital of Guangxi Medical University, Nanning, China

The original version of this article has been updated.

